# Reinforcement learning-based SDN routing scheme empowered by causality detection and GNN

**DOI:** 10.3389/fncom.2024.1393025

**Published:** 2024-04-29

**Authors:** Yuanhao He, Geyang Xiao, Jun Zhu, Tao Zou, Yuan Liang

**Affiliations:** Intelligent Manufacturing Computing Research Center, Zhejiang Lab, Hangzhou, China

**Keywords:** reinforcement learning, causal inference, graph neural network, SDN routing, quality-of-service

## Abstract

In recent years, with the rapid development of network applications and the increasing demand for high-quality network service, quality-of-service (QoS) routing has emerged as a critical network technology. The application of machine learning techniques, particularly reinforcement learning and graph neural network, has garnered significant attention in addressing this problem. However, existing reinforcement learning methods lack research on the causal impact of agent actions on the interactive environment, and graph neural network fail to effectively represent link features, which are pivotal for routing optimization. Therefore, this study quantifies the causal influence between the intelligent agent and the interactive environment based on causal inference techniques, aiming to guide the intelligent agent in improving the efficiency of exploring the action space. Simultaneously, graph neural network is employed to embed node and link features, and a reward function is designed that comprehensively considers network performance metrics and causality relevance. A centralized reinforcement learning method is proposed to effectively achieve QoS-aware routing in Software-Defined Networking (SDN). Finally, experiments are conducted in a network simulation environment, and metrics such as packet loss, delay, and throughput all outperform the baseline.

## 1 Introduction

Software-Defined Networking (SDN) routing separates the routing decision process from hardware devices, such as routers, allowing routing decisions to be made through a centralized software controller. This provides greater flexibility and stronger control capabilities. SDN routing (Tu et al., 2021, 2022; He et al., 2024) plays a crucial role in application scenarios that require high-quality network service, such as online video game and video conferencing.

The routing problem has been widely abstracted into graph theory model, where routers and links in the network are represented as nodes and edges in the graph. This model allows us to determine the optimal transmission path for data packets in the network through path selection algorithms. Early routing methods, such as distance-vector algorithms and link-state algorithms, had significant limitations in terms of computation and communication overhead, slow convergence speed, and poor network scalability. Heuristic algorithms can be used for routing optimization, but they have high computational complexity and increased the computational load on the SDN controller.

In recent years, there have been numerous studies attempting to optimize routing using machine learning techniques (Xie et al., 2019; Su et al., 2023; Teng et al., 2023; Xiao and Zhang, 2023), particularly through reinforcement learning (RL) methods. By maximizing rewards through continuous interaction with the environment, the agent is able to find optimal strategies. Causal inference can help understand the causal relationship between network events, identify cause of problems, and guide control decisions. These machine learning techniques can achieve more intelligent SDN routing, enhancing network performance and stability.

In this study, a QoS-aware network routing scheme that combines deep reinforcement learning, causal inference, and GNN is designed to improve routing performance. Real-time network state is collected by the reinforcement learning agent, which aggregates neighborhood information using CensNet (Jiang et al., 2019; Jiang et al., 2020), to obtain representations of nodes and edges as input to the deep reinforcement learning (DRL) model. The agent outputs link weights and generates routing policies based on the Dijkstra algorithm. Causal inference is used to measure the causal impact of actions on the network environment and guide the agent to explore the action space more effectively. Finally, the routing performance of the network topology is tested in a simulation environment. The innovation points of this study are mainly listed as follows:

- This study is the first to effectively combine causal inference and reinforcement learning, resulting in significant performance improvement in network routing problems.
- For QoS routing problems, causal inference is used to quantify the impact of actions, and a new reward function is designed to guide effective exploration of actions.
- Graph neural network is employed to simultaneously represent nodes and edges, which is applied to SDN network routing.
- Experiments are conducted on a network topology, and performance metrics are improved significantly, verifying the effectiveness of the proposed method.

## 2 Related work

### 2.1 Reiforcement learning

DRL-based methods (Bernardez et al., 2021; Casas-Velasco et al., 2021; Dong et al., 2021; Liu et al., 2021, 2023; Sun et al., 2022; He et al., 2023) deploy the agent within SDN controller and generate control signals based on reward feedback after interacting with the data plane. Distinguishing from supervised learning algorithms, DRL methods do not require labeled datasets and can converge to optimal policies through continuous iteration with the environment, achieving automated network operation (Sun et al., 2022). Bernardez et al. (2021) combined traffic engineering with multi-agent RL to minimize network congestion and optimize routing. Casas-Velasco et al. (2021) introduced the concept of knowledge plane into SDN and applies DRL for routing decisions. Dong et al. (2021) employed a generative adversarial network to learn domain-invariant features for DRL-based routing in various network environments. Liu et al. (2021), He et al. (2023), and Liu et al. (2023) proposed multi-agent RL approaches for hop-by-hop routing.

### 2.2 Causal inference

Causal inference is a method used to determine and quantify causal relationship by analyzing observed data and credible hypotheses, inferring causal connection between causes and effects rather than just correlation. Causal reinforcement learning is an umbrella term for RL approaches that incorporate additional assumptions or prior knowledge to analyze and understand the causal mechanism underlying actions and their consequences, enabling agents to make more informed and effective decisions. The four key applications of causal inference to RL include improving sample efficiency, enhancing generalization and knowledge transfer, eliminating spurious correlations, and studying interpretability, safety, and fairness in RL (Deng et al., 2023). Research studies such as Sontakke et al. (2021) and Huang et al. (2022) enhance sample efficiency in RL by conducting causal representation learning. Seitzer et al. (2021) improves the efficiency of the agent, exploring the action space by measuring the causal impact on the environment based on conditional mutual information. Pitis et al. (2020) explores counterfactual data by studying local independence condition in the environment, enriching the sample dataset and enhancing the generalization capability of the agent. Lu et al. (2018) eliminate decision bias of agent and improve decision accuracy by studying confounding bias in RL.

### 2.3 Graph neural network

Supervised learning algorithms (Rusek et al., 2019; Xie et al., 2019; Ferriol-Galm's et al., 2023) rely on labeled training datasets, where the model take network and traffic information as input to generate routing scheme. One major challenge of supervised learning methods is feature extraction, and the existing extraction methods generally perceive the network topological structure based on Graph Neural Network (GNN). Rusek et al. (2019) and Ferriol-Galm's et al. (2023) predicted network performance metrics (e.g., packet loss, delay, and jitter) for quality-of-service routing only through GNN.

## 3 Problem formulation

The network traffic considered in this study originates from any node and terminates at other nodes, represented as a discrete-time model (Liu et al., 2021), where traffic arrives in a predetermined time sequence. Each traffic flow is represented as a source node and a destination node. Additionally, the network topology is modeled as a bidirectional graph consisting of a collection of routers or switches and links. A DRL agent is deployed in the SDN controller, which takes network state as input and output routing control signals. The SDN controller creates routing tables and deploys them to the data plane to achieve traffic forwarding.

The objective of this study is that each traffic flow is successfully routed from the source node to the destination node, avoiding congested or failed links and maximizing the average reward for all traffic flows. It is important to note that once a routing policy is implemented on a specific traffic flow, the routing policy for that flow remains stable.

## 4 The proposed SDN routing scheme

### 4.1 System framework for SDN routing

**Figure 1:**
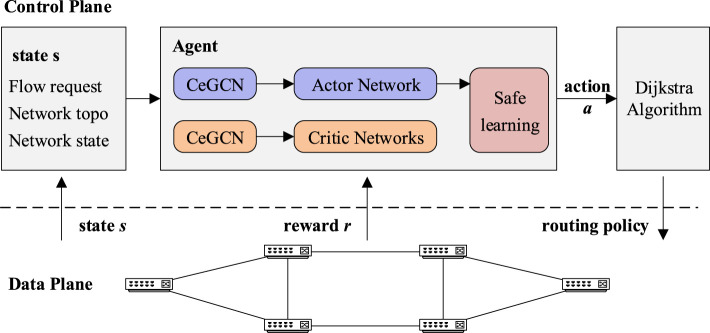
The system framework for SDN routing.

In this study, the network state and global topology are obtained by the SDN controller and used as inputs for the RL agent. The scheme utilizes soft actor-critic (Haarnoja et al., 2018) integrated with nodes and links co-embedding (Jiang et al., 2019; Jiang et al., 2020) and applies causal action influence modeling (Seitzer et al., 2021) to reward feedback, called SAC-CAI-EGCN. Routing control policies are generated as outputs and prune action through a safe learning mechanism (Mao et al., 2019). The final routing strategy is then generated using the Dijkstra algorithm and deployed to the data plane, as shown in Figure 1.

### 4.2 Design of SAC-CAI-EGCN

**Figure 2:**
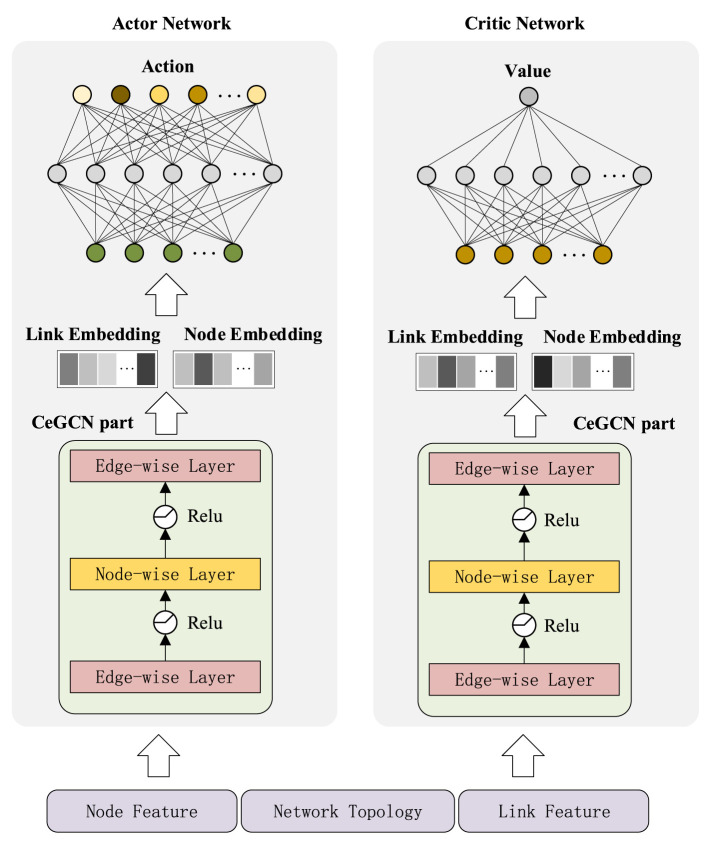
The structure of SAC-CAI-EGCN.

SAC-CAI-EGCN includes an actor net, two critic nets and two target critic nets. The structure of actor and critic nets is shown in Figure 2. CeGCN part is employed to represent nodes and links, and then, the resulting link embedding and node embedding are concatenated as input to the actor and critic nets.

#### 4.2.1 CeGCN part

To achieve simultaneous embedding of nodes and links, a three-layer network structure called CeGCN is designed, as shown in Figure 2. It consists of two edge-wise layers and a node-wise layer. The node-wise layer updates node embedding by combining the updated link embedding with propagation process referring to Equation (1). The edge-wise layer updates link embedding based on the input data with information propagation referring to Equation (2).

The node-wise propagation process of node features is shown in Equation (1).


(1) $$\begin{array}{l} \begin{array}{c} H_{v}^{\left(l+1\right)}=\sigma \left(T\Phi \left(H_{e}^{\left(l\right)}P_{e}\right)T^{T}⊙{\tilde{A}}_{v}H_{v}^{\left(l\right)}W_{v}\right) \end{array} \end{array}$$

The edge-wise propagation process of edge features is shown in Equation (2).


(2) $$\begin{array}{l} \begin{array}{c} H_{e}^{\left(l+1\right)}=\sigma \left(T^{T}\Phi \left(H_{v}^{\left(l\right)}P_{v}\right)T⊙{\tilde{A}}_{e}H_{e}^{\left(l\right)}W_{e}\right) \end{array} \end{array}$$

In which, *T* is a transformation matrix and *T*_*i, m*_ represents whether node *i* connects edge *m*. Φ denotes the diagonalization operation of a matrix. *P*_*e*_ and *P*_*v*_, respectively, represent the learnable weights of edge and node feature vectors. ⊙ denotes the element-wise product operation. *W*_*v*_ is the network parameter in the node-wise propagation process, so as *W*_*e*_.

In Equations (1, 2), ${\tilde{A}}_{e}$ and ${\tilde{A}}_{v}$ are calculated as Equation (3). *A*_*i*_ represents the adjacency matrix of nodes or edges, and *i* represents the node or edge. *I*_*N*_*i*__ is an identity matrix and *D*_*i*_ is the diagonal degree matrix of *A*_*i*_+*I*_*N*_*i*__.


(3) $$\begin{array}{l} \begin{array}{c} {\tilde{A}}_{i}=D_{i}^{-\frac{1}{2}}\left(A_{i}+I_{N_{i}}\right)D_{i}^{-\frac{1}{2}} \end{array} \end{array}$$

#### 4.2.2 DRL model

The agent is trained based on a quadruple data structure < *S, A, R, S*′>, which is defined in detail as follows:

- State *S*: the current state mainly includes (1) the representation of nodes and links generated by the CeGCN part; (2) the topology of network; and (3) the flow request. Specifically, the raw features for representation include the remaining available bandwidth and packet loss rate of each link, the number of flows, and the total size of data packets of each node.
- Action *A*: the weights of links in the network which are decimals and belong to the interval (0, 1].
- Reward *R*: for comprehensive calculation of packet loss rate, delay, and throughput, a reward function is designed as follows:
(4) $$\begin{array}{l} \begin{array}{c} r_{t}=-\gamma_{1}x_{t}-\gamma_{2}y_{t}+\gamma_{3}z_{t}+\gamma_{4}r_{t}^{cai} \end{array} \end{array}$$
(5) $$\begin{array}{l} \begin{array}{c} r_{t}^{cai}=\frac{1}{N}\overset{N}{\underset{j}{\sum }}C^{j}\left(s_{t}\right) \end{array} \end{array}$$
(6) $$\begin{array}{l} \begin{array}{c} C^{j}\left(s_{t}\right)=I\left(S_{j}^{\prime };A|S=s_{t}\right)={𝔼}_{a\sim \pi }\left[{\text{D}}_{\text{KL}}\left(P_{S_{j}^{\prime }|s_{t},a}||P_{S_{j}^{\prime }|s_{t}}\right)\right] \end{array} \end{array}$$
  In Equation (4), let *x*_*t*_ represents the packet loss rate, *y*_*t*_ represents the delay, and *z*_*t*_ represents throughput at *t* time slot, respectively. γ_1_, γ_2_, γ_3_, and γ_4_, respectively, represent the weight of packet loss rate, delay, throughput, and causal influence in the reward function. In this study, the reward function assigns weights to prioritize packet loss rate, delay, and throughput in the following order: γ_1_, γ_2_, γ_3_, and γ_4_ are assigned to be 2, 1.5, 1, and 1, respectively. In Equations (5, 6), $S_{j}^{\prime }$ represents the *j*-th component of *S*′, *D*_*KL*_ denotes the KL divergence, and *C*^*j*^(*s*) quantifies the causal influence of action *A* on $S_{j}^{\prime }$ given the state *S* = *s*_*t*_.
  In specific scenarios, packet loss rate, delay, and throughput are not on the same scale, so normalization is required. The normalization operation for packet loss rate and delay is as follows:
(7) $$\begin{array}{l} \begin{array}{c} x_{t}=\frac{x_{t}}{{\bar{x}}_{t}} \end{array} \end{array}$$
(8) $$\begin{array}{l} \begin{array}{c} y_{t}=\frac{y_{t}-y_{base}}{{\bar{y}}_{t}} \end{array} \end{array}$$
  Between Equations (7) and (8), *y*_*base*_ represents the average delay of all links in the network, which is set to be 5 ms. ${\bar{x}}_{t}$ and ${\bar{y}}_{t}$ denote the average loss rate and delay of the recent flows with the same source and destination as the flow at time slot *t*, which are approximated by the following equation.
(9) $${\bar{m}}_{t}=\left\{ \begin{array}{l} m_{t},\;t=1\\ \epsilon \cdot {\bar{m}}_{t-1}+(1-\epsilon )\cdot m_{t},\;t\geq 2 \end{array}\right.$$
  ϵ is a constant used to control the update rate of Equation (9). In this study, ϵ is 0.8.
  For the throughput, after normalizing according to the bandwidth requirement *z*_*demand*_, it needs the logarithmic change. The process is presented as Equation (10):
(10) $$\begin{array}{l} \begin{array}{c} z_{t}=log\left(\frac{z_{t}}{z_{demand}}+b\right) \end{array} \end{array}$$
  In Equation (10), in order to avoid abnormal values in the log operation, the parameter *b* = 0.5 is added.
- State *S*′: after executing action *A*, state *S*′ is acquired and it contains the same type of information as state *S*.

The experience of interacting with the environment is stored in the replay buffer and sampled by prioritized experience replay. The policy π_θ_(*s*) is updated by the temporal difference method, in which θ represents the parameter of policy network. The loss function of actor net and critic net is as follows:


(11) $$\begin{array}{l} \begin{array}{c} {\tilde{a}}_{t}=f_{\theta ,\epsilon_{t}\sim 𝒩}\left(\epsilon_{t};s_{t}\right) \end{array} \end{array}$$

(12) $$\begin{array}{l} \begin{array}{c} L_{\pi }\left(\theta \right)={𝔼}_{s_{t}\sim R}\left[\alpha log\pi_{\theta }\left({\tilde{a}}_{t}|s_{t}\right)-\underset{j=1,2}{min}Q_{\omega_{j}}\left(s_{t},{\tilde{a}}_{t}\right)\right] \end{array} \end{array}$$

(13) $$\begin{array}{l} \begin{array}{c} y_{t}=r_{t}+\lambda \left[\underset{j=1,2}{min}Q_{\omega_{j}^{-}}\left(s_{t+1},a_{t+1}\right)-\alpha log\pi \left(a_{t+1}|s_{t+1}\right)\right] \end{array} \end{array}$$

(14) $$\begin{array}{l} \begin{array}{cc} L_{Q}\left(\omega \right) & =E_{\left(s_{t},a_{t},r_{t},s_{t+1}\right)\sim R}\left[\frac{1}{2}{\left(Q_{\omega }\left(s_{t},a_{t}\right)-y_{t}\right)}^{2}\right] \end{array} \end{array}$$

As shown in the above equations, ϵ_*t*_ is a random noise variable sampled from the unit gaussian distribution 𝒩. ${\tilde{a}}_{t}$ is obtained through the reparameterization trick of Equation (11). The loss of actor net is calculated based on Equation (12). *a*_*t*+1_ is obtained by π_θ_(·|*s*_*t*+1_), and the loss of any critic net is calculated by Equations (13, 14).

As shown in Algorithm 1, lines 1–3 are to initialize actor net, two critic nets, two target critic nets, and replay buffer. Lines 4–10 collect experience, line 8 calculates the causal action influence $r_{t}^{cai}$, then lines 9–10 calculate reward *r*_*t*_ and store them to the replay buffer R. Line 13 updates two critic nets *Q*_ω_1__(*s, a*), *Q*_ω_2__(*s, a*), and then lines 14–15 update actor net π_θ_(*s*). Moreover, line 17 softly synchronizes parameter to the two target critic nets $Q_{\omega_{1}^{-}}\left(s,a\right)$, $Q_{\omega_{2}^{-}}\left(s,a\right)$.

**Algorithm 1:** SAC-CAI-EGCN routing algorithm.

**Input:** Replay buffer R, Actor Network π_θ_(*s*), Critic Networks *Q*_ω_1__(*s, a*), *Q*_ω_2__(*s, a*), Target Critic Networks $Q_{\omega_{1}^{-}}\left(s,a\right)$, $Q_{\omega_{2}^{-}}\left(s,a\right)$, Entropy regularization coefficient α, Parameter synchronization weighted τ
**Output:** Actor Net π_θ_(*s*)
1: Randomly initialize network parameters θ, ω_1_, ω_2_ of Actor network and two Critic Networks
2: Copy the same parameters $\omega_{1}\to \omega_{1}^{-},\omega_{2}\to \omega_{2}^{-}$ to initialize Target Critic Networks
3: Initialize the replay buffer R
4: **for** Episode = 1, E **do**
5: **for** *t* in Trajectory **do**
6: obtain state *s*_*t*_ from the environment
7: execute action *a*_*t*_, then get new state *s*_*t*+1_
8: calculate $r_{t}^{cai}$ according to Equations (5, 6)
9: calculate reward *r*_*t*_ according to Equation (4)
10: add experience < *s*_*t*_, *a*_*t*_, *r*_*t*_, *s*_*t*+1_> into R
11: **for** Training rounds = 1, K **do**
12: sample mini batch *B* from R by Prioritized Experience Replay
13: calculate loss with Equations (13, 14) and update Critic Networks *Q*_ω_1__(*s, a*), *Q*_ω_2__(*s, a*) by Adam optimizer
14: Sample action by reparameterization trick based on Equation (11)
15: calculate loss with Equation (12) and update Actor network by Adam optimizer
16: update Entropy regularization coefficient α
17: update Target Critic Networks by: $\tau \omega_{i}+\left(1-\tau \right)\omega_{i}^{-}\to \omega_{i}^{-},i\in \left\{1,2\right\}$
18: **end for**
19: **end for**
20: **end for**

#### 4.2.3 Safe learning mechanism for routing

**Figure 3:**
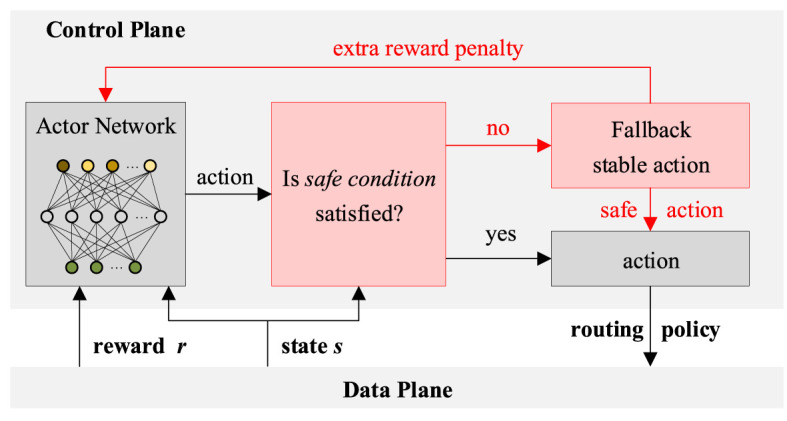
The safe learning mechanism for SDN routing.

To prevent the degradation of performance caused by unsafe strategies, such as passing through failure or heavily congested links, a safe learning mechanism is designed. As shown in Figure 3, for each decision-making process, the control plane will determine whether the *safe condition* is met. For the current action and status *s*, the safe condition containing the following two items needs to be met simultaneously: (1) not passing through failure links and (2) not going through heavily congested links. If the safe condition is satisfied, the action will be output directly. If not, a fallback stable action will be output. Specifically, the weights of failure links or heavily congested links will be modified to the maximum value, which is 1. At the same time, an extra reward penalty will be fed back to guide the actor net to generate safer routing policies.

## 5 Experiments

### 5.1 Simulation setup

A public network topology is used, namely, GEANT2. It has 24 nodes and 37 bidirectional links. In the simulation environment, most of the links have a data rate of 10 Mbps, while there is two bottleneck links in GEANT2 with a data rate of 2.5 Mbps. Overall, 10% packet loss is added to each bottleneck link. Moreover, each link has a transmission delay of 5 ms.

In this study, the shortest path routing (SPR) is selected as the typical method for comparison, which only calculates the shortest hop number without considering the network state. The other one is SAC with causal action influence modeling called SAC-CAI.

### 5.2 Experiment results

#### 5.2.1 Performance under given network

For a given network topology, the starting and ending nodes of flows are generated randomly, and the exact same traffic is used to test the three methods. For the GEANT2 network, the duration of flows is set to be 35 time slots, the global steps for the three methods are, respectively, set to be 30,000, 100,000, and 100,000.

Table 1 presents a comparison of three methods in terms of performance metrics including packet loss rate, latency, and throughput under the GEANT2 network topology. From the model reward curves, SAC-CAI and SAC-CAI-EGCN converge ~100,000 steps, while SPR exhibits congestion and latency at ~30,000 steps, so the SPR method only runs 30,000 steps. SAC-CAI-EGCN outperforms SPR and SAC-CAI significantly in all metrics under the same network topology, flows, and traffic intensity. First, the superior performance of SAC-CAI over SPR indicates the positive impact of causal inference in guiding action exploration for network routing. Second, SAC-CAI-EGCN exploits link and node co-embedding to effectively aggregate neighborhood features, thereby enhancing network routing performance in comparison with SAC-CAI.

**Table 1:** Performance results under GEANT2 network.

**Method**	**Avg.lossRate**	**Avg.delay (ms)**	**Avg.thrpt (Kbps)**
SPR	0.269	302.811	1025.295
SAC-CAI	0.090	108.107	1264.046
SAC-CAI-EGCN	**0.089**	**106.653**	**1266.710**

The bold values indicate optimal performance in the current column.

#### 5.2.2 Performance under different traffic intensities

**Figure 4:**
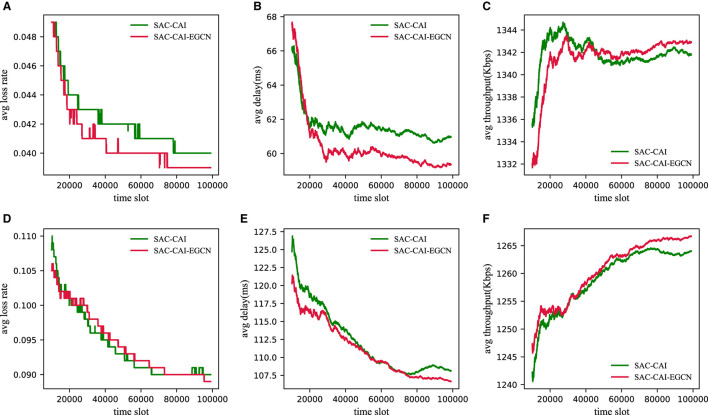
Performance results of three methods under different traffic intensities (light-load and heavy-load) in the GEANT2 network. **(A)** Loss rate under light-load. **(B)** Delay under light-load. **(C)** Throughput under light-load. **(D)** Loss rate under heavy-load. **(E)** Delay under heavy-load. **(F)** Throughput under heavy-load.

To investigate the performance of SAC-CAI-EGCN, SAC-CAI, and SPR under different traffic intensities, an additional experiment with 25 time slot (light-load) flows was conducted. However, due to the poor performance of SPR and its significant difference in data scale compared with the other two methods, only the experimental results of SAC-CAI-EGCN and SAC-CAI are presented, as shown in Figure 4. First, as the traffic intensity increases, the packet loss rate and latency increase, while the throughput decreases. Second, from light to heavy traffic intensity, SAC-CAI-EGCN demonstrates superior performance in terms of packet loss rate, latency, and throughput compared with SAC-CAI.

## 6 Conclusion

In this study, based on action influence quantification and GNN a reinforcement learning method is proposed, enabling efficient SDN routing. Experimental results on publicly available network topology and different traffic intensities demonstrate significant improvement in QoS metrics, such as packet loss rate, latency, and throughput compared with baselines. This validates the effectiveness of SAC-CAI-EGCN in quantifying the causal impact of actions on the environment and simultaneously embedding edges and node features, guiding the generation of efficient SDN routing policies. In the future, we will continue exploring the application of causal reinforcement learning in improving network service quality, such as leveraging counterfactual data augmentation to improve sample efficiency and addressing confounding bias in RL.

## Data availability statement

Publicly available datasets were analyzed in this study. This data can be found at: https://www.cedefop.europa.eu/nl/news/geant2-world-beating-infrastructure-research.

## Author contributions

YH: Conceptualization, Data curation, Methodology, Resources, Software, Validation, Writing – original draft, Writing – review & editing. GX: Formal analysis, Software, Supervision, Writing – review & editing. JZ: Visualization, Writing – review & editing. TZ: Writing – review & editing. YL: Writing – review & editing.
